# Effects of action observation and motor imagery of walking on the corticospinal and spinal motoneuron excitability and motor imagery ability in healthy participants

**DOI:** 10.1371/journal.pone.0266000

**Published:** 2022-04-18

**Authors:** Naotsugu Kaneko, Atsushi Sasaki, Hikaru Yokoyama, Yohei Masugi, Kimitaka Nakazawa

**Affiliations:** 1 Department of Life Sciences, Graduate School of Arts and Sciences, The University of Tokyo, Tokyo, Japan; 2 Japan Society for the Promotion of Science, Tokyo, Japan; 3 School of Health Sciences, Tokyo International University, Saitama, Japan; Federation University Australia, AUSTRALIA

## Abstract

Action observation (AO) and motor imagery (MI) are used for the rehabilitation of patients who face difficulty walking. Rehabilitation involving AO, MI, and AO combined with MI (AO+MI) facilitates gait recovery after neurological disorders. However, the mechanism by which it positively affects gait function is unclear. We previously examined the neural mechanisms underlying AO and MI of walking, focusing on AO+MI and corticospinal and spinal motor neuron excitability, which play important roles in gait function. Herein, we investigated the effects of a short intervention using AO+MI of walking on the corticospinal and spinal motor neuron excitability and MI ability of participants. Twelve healthy individuals participated in this study, which consisted of a 20 min intervention. Before the experiment, we measured MI ability using the Vividness of Movement Imagery Questionnaire-2 (VMIQ-2). We used motor evoked potential and F-wave measurements to evaluate the corticospinal and spinal motor neuron excitability at rest, pre-intervention, 0 min, and 15 min post-intervention. We also measured corticospinal excitability during MI of walking and the participant’s ability to perform MI using a visual analog scale (VAS). There were no significant changes in corticospinal and spinal motor neuron excitability during and after the intervention using AO+MI (p>0.05). The intervention temporarily increased VAS scores, thus indicating clearer MI (p<0.05); however, it did not influence corticospinal excitability during MI of walking (p>0.05). Furthermore, there was no significant correlation between the VMIQ-2 and VAS scores and changes in corticospinal and spinal motor neuron excitability. Therefore, one short intervention using AO+MI increased MI ability in healthy individuals; however, it was insufficient to induce plastic changes at the cortical and spinal levels. Moreover, the effects of intervention using AO+MI were not associated with MI ability. Our findings provide information about intervention using AO+MI in healthy individuals and might be helpful in planning neurorehabilitation strategies.

## 1. Introduction

Action observation (AO) and motor imagery (MI) are used for the rehabilitation of patients with neurological disorders. AO can be defined as “the perception of other’s actions” [1,2], whereas MI can be defined as “the mental simulation or rehearsal of a movement without any motor output” [3]. Both AO and MI are motor simulations that recruit neural systems related to observed and imagined movements, without action execution and muscle contraction. For example, AO and MI facilitate corticospinal excitability [1,4]. The modulation of AO and MI-induced neural activity contributes to the improvement of motor function. AO, MI, and AO combined with MI (AO+MI) of walking have been used as gait rehabilitation methods for patients who face difficulty in walking. Rehabilitation involving the aforementioned techniques facilitates gait improvement after neurological disorders, such as stroke and Parkinson’s disease [5–10]. However, the mechanism by which such rehabilitation positively affects gait improvement is unclear.

The modulation of neural activity during AO, MI, and AO+MI of walking is considered a factor in gait improvement. In healthy individuals, both AO and MI of walking activate the premotor cortex and the supplementary motor area involved in actual walking [11–13]. In addition to the motor cortex, AO of walking facilitates corticospinal excitability [14] and modulates spinal reflexes [15,16], whereas MI of walking does not change corticospinal excitability [17]. Our recent studies have focused on neural activities underlying AO+MI of walking [13,16,18]. The facilitation of corticospinal and spinal motor neuron excitability during AO+MI of walking was greater than that during AO alone [16,19]. Furthermore, AO+MI induces higher activation of the entire cortex than AO alone [13].

Despite several studies on the modulation of neural activity during AO, MI, and AO+MI of walking, the process by which these modalities alter neural activity post intervention is unclear. A recent review claimed that AO+MI interventions might provide more effective methods for motor improvement and learning than those using AO or MI alone [20]. Increases in corticospinal excitability and cortical activity during AO+MI are greater than those during AO or MI alone [21–28]. Therefore, AO+MI induces neural plastic changes more effectively than AO and MI alone.

We focused on interventions using AO+MI of walking in healthy individuals. Our first purpose was to investigate the effects of the intervention on corticospinal and spinal motor neuron excitability. These structures play a crucial role in controlling movement and gait: the corticospinal tract is the major pathway transmitting a motor command from the motor cortex to the spinal cord, whereas the spinal motoneurons are the final common pathway connecting the spinal cord to the muscles. We also investigated the relationship between changes in excitability and MI ability, as assessed by the Vividness of Movement Imagery Questionnaire-2 (VMIQ-2) [29], because individual differences in MI ability could vary the effect of the intervention. We hypothesized that the intervention using AO+MI of walking facilitates corticospinal and spinal motor neuron excitability and that this facilitation is greater in participants with higher MI ability.

We also focused on the effects of AO+MI intervention on MI ability. Our second purpose was to investigate the mechanism by which the intervention affects the ability to perform MI of walking. The AO component of AO+MI likely works as an external visual scaffolding of MI [30]. Thus, we hypothesized that the ability to perform MI of walking increases post-intervention. Our hypotheses were tested using a visual analog scale (VAS) [31], and we measured the corticospinal excitability during MI of walking [17].

## 2. Material and methods

### 2.1. Participants

We enrolled 18 participants (13 men and 5 women, age: 25.1 ± 1.9 years (23–29 years), height: 169.6 ± 10.1 cm (150–187 cm), and weight: 63.4 ± 9.1 kg (42–80 kg), as mean ± SD with the range in brackets), each without a history of neurological disorders. All participants provided written informed consent, and the experimental procedures were approved by the local ethics committee of the University of Tokyo. The study was performed in accordance with the tenets of the Declaration of Helsinki (1964).

### 2.2. Preparation for the intervention

**Fig 1:**
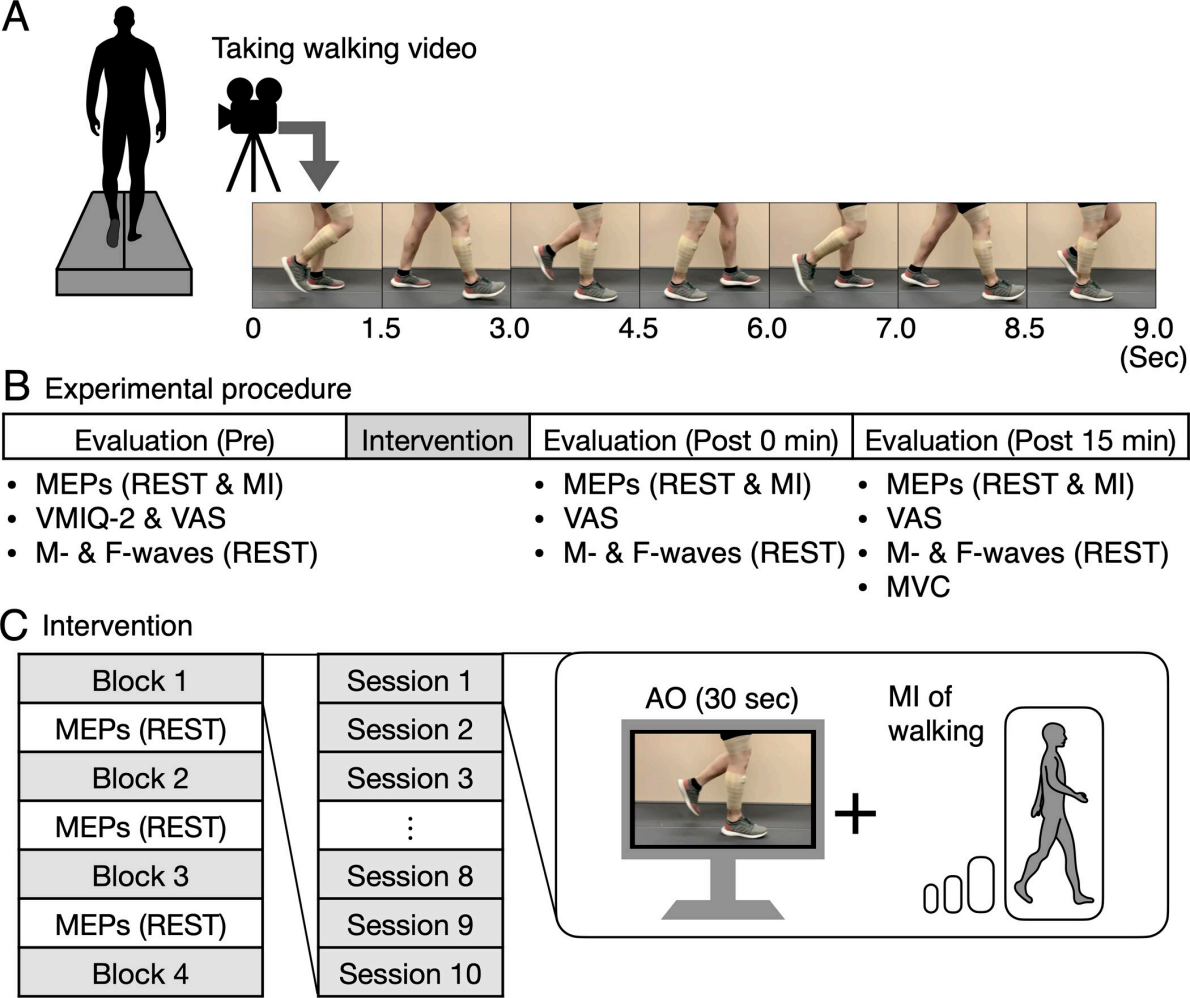
Preparation for the intervention, study design, and experimental procedure. A video of a healthy man walking is captured for action observation (AO), and examples of 9 s of the walking video are displayed (A). The experiment consists of evaluations and interventions (B). Before and after the intervention, motor evoked potentials (MEPs), F-waves, and M-waves are recorded when the participants relaxed (REST). In addition, MEPs are recorded when the participants performed motor imagery (MI) of walking. The intervention consists of four blocks and three evaluation sessions, with each block consisting of 10, 30-s sessions (C). For each block, a walking video is provided for 30 s, and the participants are asked to observe the video and imagine walking (i.e., AO+MI). For each session, MEPs are recorded when the participants relaxed (REST). At the end of the experiment, maximal voluntary contraction (MVC) is recorded for each recorded muscle.

Prior to the experiment, we captured a video of a healthy man (age: 25 years, height: 180 cm, and weight: 80 kg) walking for 2 min at a speed of 1.0 m/s on a treadmill (Bertec, Columbus, OH, USA) (Fig 1A). We used 30 s of the walking video. The walker did not participate in the experiment that investigated the effects of the intervention using the video.

### 2.3. Electromyographic recording

Electromyographic (EMG) signals were recorded from the right tibialis anterior (TA) and soleus (SOL) muscles. We placed bipolar Ag/AgCl surface electrodes (Vitrode F-150S; Nihon Kohden, Tokyo, Japan) over each muscle belly with at least 1-cm-wide separation after cleaning the skin with alcohol. A ground reference electrode was placed around the right knee. The EMG signals were amplified (×1,000) and filtered with a band-pass filter between 15 Hz and 1 kHz using a bio-amplifier system (MEG-6108; Nihon Kohden, Tokyo, Japan). The analog signals were digitized at a sampling rate of 4 kHz using an analog-to-digital converter (Powerlab/16SP, AD Instruments, Castle Hill, Australia).

### 2.4. Stimulus settings

Transcranial magnetic stimulation (TMS) was applied over the primary motor cortex using a magnetic stimulator (Magstim 200, Magstim Co., Whitland, UK) that delivered monophasic pulses with a posterior-anterior current direction through a double-cone coil (external diameter: 110 mm; Magstim Co., Whitland, UK). We determined the optimal coil position or the hotspot for each of the SOL muscles on observing the largest motor evoked potential (MEP) amplitude elicited from the SOL muscle. The hotspot was used as a target using a TMS neuronavigation system (BrainSight, Rogue Research, Montreal, QC, Canada). The neuronavigation system enabled maintaining a correct coil position over the hotspot throughout the experiment. The resting motor threshold (RMT) at the hotspot for the SOL muscle was based on previously established guidelines [32] and was defined as the minimum TMS intensity that evoked MEPs with peak-to-peak amplitudes ≥50 μV in the SOL muscle at rest in at least five of 10 successive trials. RMTs for the SOL muscle corresponded to 40–61% of the maximum stimulator output (mean ± SD = 49.4 ± 6.5%). The stimulation intensity for TMS was set to 110% of the RMT (1.1 RMT) for evaluating the effects of the intervention on corticospinal excitability.

Electrical stimulation was delivered to the tibial nerve using a constant-current electrical stimulator (DS7A, Digitimer, Welwyn Garden City, UK). The stimulus pulse duration was set to 1 ms. For electrical stimulation of the tibial nerve, an anode electrode measuring 50 × 50 mm (StimTrode, Axelgaard, Fallbrook, USA) was placed over the patella. A cathode electrode measuring 18 × 36 mm (Vitrode F-150S; Nihon Kohden, Tokyo, Japan) was placed over the posterior tibial nerve at the popliteal fossa. The electrodes were fixed in position with an adhesive tape. We determined the intensity to induce an M-wave with the maximum amplitude (i.e., Mmax) by visual inspection with an oscilloscope. Moreover, we used a stimulation intensity set to 20% above the intensity to induce Mmax for recording an F-wave (mean ± SD = 47.4 ± 13.3 mA). We did not measure the F-wave for one participant who could not tolerate the pain of stimulation.

### 2.5. Experimental procedure

All participants were seated in a chair placed 1.5 m away from a 32-inch screen (697.7 × 392.3 mm, Multisync V321, NEC, Tokyo, Japan). They were requested to keep their bodies relaxed throughout the experiment. At the beginning of the experiment, we assessed their ability to perform visual and kinesthetic MI of movements using the VMIQ-2 [29]. The VMIQ-2 was used to assess levels of vividness with external visual imagery, internal visual imagery, and kinesthetic imagery of 12 motor tasks (e.g., running downhill). The participants had to rate the vividness on a five-point scale (1 = perfectly vivid and as clear as normal vision to 5 = no image at all), which provided the VMIQ-2 score. The VMIQ-2 scores demonstrated comprehensive MI ability [29].

Fig 1B and 1C depict the experimental procedures for the intervention and evaluation. We recorded 15 MEPs in the REST and MI conditions, before intervention and at 0 min and 15 min post-intervention. In the REST condition, we requested that the participants relax and not imagine anything. In the MI condition, they were asked to kinesthetically imagine that they were walking without performing voluntary contraction. We provided the following instruction: “*Please imagine kinesthetically that you are walking without performing voluntary muscle contraction*.” We confirmed that the participants could perform the MI using a VAS [31]. After completing the MI, they were asked to make a mark on a 10-cm-long VAS line on paper, which provided the VAS score. The right and left extremities were labeled “None at all” (0 cm) and “Perfectly clear and vivid” (10 cm), respectively. VAS scores demonstrated the vividness of the MI of walking. Subsequently, we recorded 20 F-waves and M-waves in the REST condition.

The intervention consisted of four blocks. For each block, we displayed a 30-s video of walking 10 times. Thus, except for the break and evaluation, the intervention using AO+MI took 20 min, which was also based on previous studies in healthy individuals [33,34] and on clinical studies [5–10]. For the intervention, the participants were instructed to observe the walker’s right leg and kinesthetically imagine that they were walking similarly without performing voluntary contraction. We provided the following instruction: “P*lease observe his right leg and imagine kinesthetically that you are walking according to the observed stance and swing phases of walking without performing voluntary contraction*.” The same instructions were provided to all participants. EMG signals were recorded to confirm the absence of voluntary contractions in the recorded muscles. We recorded 15 MEPs in the REST condition between the blocks to investigate the time course of the intervention effects.

Post-intervention, we obtained 15 MEPs in the REST and MI conditions, F-wave, and M-wave in the REST condition at 0 min and 15 min as well as that before the intervention. At the end of the experiment, we recorded EMG signals for the maximum voluntary contraction (MVC) of the recorded muscles. The participants were requested to contract each muscle at maximal strength against manual resistance and hold the position for 3 s, while the experimenter held their ankle to prevent movement.

### 2.6. Data and statistical analyses

The peak-to-peak MEP amplitudes in the recorded muscles were calculated offline using a custom-written script in MATLAB (2019b, The MathWorks Inc., Natick, MA, USA). We averaged the MEP amplitudes in the REST and MI conditions for individual participants at each time point (i.e., before intervention, 0 min, and 15 min post-intervention, after each block during the intervention). The average MEP amplitudes in the REST condition obtained after and during the intervention were normalized as the percentage of the average amplitudes recorded before the intervention. The MEP amplitudes in the MI condition were normalized as a percentage of those in the REST condition at each time point. The peak-to-peak amplitudes of M-waves and F-waves in the SOL muscle were calculated and averaged for each participant. For the F-waves, a detection threshold of 50 μV was used to define the detectable response, that is, the responses that were larger than the threshold [35–37]. The persistence of the F-waves was calculated from the percentage of the number of the detectable responses to the number of stimuli (i.e., 20 times). Four participants who did not show any single detectable response were excluded from the further analysis of F-wave amplitude and persistence. Then, we obtained the maximum amplitudes of the M-waves (i.e., Mmax) at each time point. The amplitudes of the F-waves were normalized to Mmax, which provided the F/M. The EMG root mean square (RMS) values of a 50 ms window before recording the MEP and F-waves were defined as the background EMG activity for each muscle. MEPs and F-waves with a non-normalized background EMG greater than 10 μV were excluded from statistical analyses. As a result, three MEPs out of 2430 and eight F-waves out of 840 were removed. Background EMG was normalized according to the EMG activity for MVC. MVC in each muscle was calculated as the RMS value of a 50 ms window while the participants performed MVC.

All statistical analyses were performed using the SPSS Statistics ver. 25 (IBM Corp., Chicago, IL, USA). First, we performed statistical analyses to investigate the effect of the intervention on corticospinal excitability in the TA and SOL muscles in the REST condition. Furthermore, we conducted non-parametric tests, because the Shapiro-Wilk test demonstrated that the normalized MEP amplitudes were not normally distributed. We conducted the Friedman test, a non-parametric equivalent for a repeated-measure analysis of variance (rm-ANOVA), to compare the normalized MEP amplitudes before, during (i.e., after the first block [Int 5], second block [Int 10], and third block [Int 15]), and after the intervention (i.e., 0 min and 15 min after the intervention). Friedman tests were also conducted to compare the normalized background EMG activity during and after the intervention. On observing a significant effect from the Friedman test, we performed Wilcoxon signed-rank tests as post-hoc tests. Spearman’s correlation analyses were performed to investigate the correlations between the VMIQ-2 scores and MEP amplitude changes during and after the intervention.

Second, we performed statistical analyses to investigate the effect of the intervention on corticospinal excitability during MI of walking in the TA and SOL muscles. Friedman tests were conducted to compare the normalized MEP amplitudes and background EMG activity between the REST and MI conditions at each time point (i.e., before intervention, 0 min, and 15 min post-intervention) and VAS scores after performing walking MI at each time point. Upon obtaining significant effects from the Friedman tests, we performed Wilcoxon signed-rank tests for multiple comparisons using post-hoc tests. Spearman’s correlation analyses were performed to investigate the correlations between the VAS and VMIQ-2 scores and changes in MEP amplitudes in the MI condition.

Third, we also performed statistical analyses to investigate the effects of the intervention on the Mmax and F-waves in the SOL muscle. The Shapiro-Wilk tests demonstrated that Mmax and F/M were normally distributed. One-way rm-ANOVAs were conducted to compare the amplitudes of Mmax and F/M before and after the intervention (i.e., before, 0 min, and 15 min post-intervention). We performed paired t-tests for multiple comparisons using post-hoc tests on obtaining significant effect in the rm-ANOVAs. In case of a significant violation of the assumption of sphericity (Mauchly’s test, p<0.05), we conducted Greenhouse-Geisser adjustments to the degrees of freedom. The Shapiro-Wilk test revealed that the persistence of F-wave and the normalized background EMG activity were not normally distributed. Friedman tests were conducted to compare the persistence of F-wave and the normalized background EMG activity in the REST condition at each time point (i.e., before intervention, 0 min, and 15 min post-intervention). We performed Wilcoxon signed-rank tests for multiple comparisons using post-hoc tests on obtaining significant effects in the Friedman test. Spearman’s correlation analyses were performed to investigate the correlations between the VMIQ-2 scores and changes in F/M post-intervention.

The significance level was set to 0.05 in all statistical tests. We used the Bonferroni method to correct the p-values for post-hoc tests for multiple comparisons. We also used the Benjamini-Hochberg false discovery rate (FDR) method to adjust the p-values for multiple correlation analyses. The eta squared values for Friedman and ANOVA tests, r values for Wilcoxon signed-rank tests, and d values for paired t-tests were calculated as the effect size indices [38–40]. The thresholds for interpreting the eta squared values were set to 0.01, 0.06, and 0.14 for small, medium, and large, respectively, whereas those for interpreting the r values were set at 0.1, 0.3, and 0.5, for small, medium, and large, respectively (Cohen, 1988; Morse, 1999; Rosenthal et al., 1994). Data are presented as the mean ± SD.

## 3. Results

### 3.1. MEP, F-wave, and Mmax

**Fig 2:**
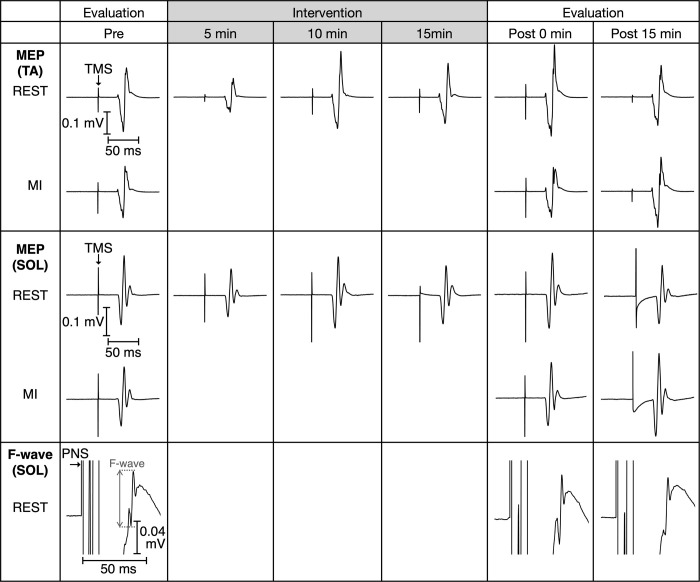
Example waveforms of motor evoked potential and F-wave recorded from one participant. The figure depicts mean motor evoked potential (MEP) waveforms in the tibialis anterior (TA) and soleus (SOL) muscles recorded before intervention, 0 min, and 15 min after the intervention in the REST and MI conditions. The mean MEP waveforms in the TA and SOL muscles in the REST condition are recorded during the intervention (i.e., after the first block [5 min], second block [10 min], and third block [15 min]). The mean waveforms of the F-wave in the SOL muscle recorded before intervention, 0 min, and 15 min after the intervention in the REST condition are displayed.

Fig 2 represents the mean waveforms of the MEP and F-waves recorded from a participant. Table 1 summarizes the average non-normalized and normalized amplitudes of MEP in the REST condition before the intervention; after the first, second, and third blocks during the intervention; and at 0 min and 15 min post-intervention. Table 2 summarizes the average non-normalized and normalized amplitudes of MEP in the REST and MI conditions before intervention and at 0 min and 15 min post-intervention. Table 3 outlines the average non-normalized amplitudes of F-wave, Mmax, F/M, and persistence in the REST condition before intervention, 0 min, and 15 min post-intervention.

**Table 1:** Average non-normalized and normalized (% of Pre) amplitudes of MEP with SDs in the rest condition before intervention (Pre), after first block (Int 5), after second block (Int 10), after third block (Int 15), immediately after intervention (Post 0), and 15 min after intervention (Post 15).

		Non-normalized amplitude (mV)			Normalized amplitude (%)		
MEP	Pre	0.183	±	0.124	100	±	0
(TA)	Int 5	0.167	±	0.150	94.8	±	35.5
	Int 10	0.152	±	0.136	85.9	±	35.1
	Int 15	0.176	±	0.132	103.1	±	42.2
	Post 0	0.191	±	0.153	104.2	±	34.2
	Post 15	0.181	±	0.113	104.1	±	38.2
MEP	Pre	0.1060	±	0.0574	100	±	0
(SOL)	Int 5	0.0972	±	0.0605	94.2	±	30.5
	Int 10	0.1012	±	0.0886	91.6	±	36.5
	Int 15	0.1030	±	0.0919	95.3	±	40.2
	Post 0	0.1130	±	0.0732	107.7	±	42.2
	Post 15	0.1051	±	0.0977	97.8	±	44.5

MEP, motor evoked potential; TA, tibialis anterior; SD, standard deviation; and SOL, soleus.

**Table 2:** Average non-normalized and normalized (% of REST) amplitudes of MEP with SDs in the REST and MI conditions before intervention (Pre), immediately after intervention (Post 0), and 15 min after intervention (Post 15).

		Non-normalized amplitude (mV)						Normalized amplitude		
		Rest			MI			MI (%REST)		
MEP	Pre	0.183	±	0.124	0.187	±	0.132	108.6	±	415
(TA)	Post 0	0.191	±	0.153	0.199	±	0.173	109.2	±	37.5
	Post 15	0.181	±	0.113	0.187	±	0.121	110.0	±	36.8
MEP	Pre	0.106	±	0.057	0.108	±	0.064	107.7	±	36.7
(SOL)	Post 0	0.113	±	0.073	0.106	±	0.064	97.8	±	24.8
	Post 15	0.105	±	0.098	0.119	±	0.105	107.7	±	29.3

MEP, motor evoked potential; TA, tibialis anterior; MI, motor imagery; SD, standard deviation; and SOL, soleus.

**Table 3:** Average non-normalized amplitudes of F-wave and Mmax, F/M, and persistence of F-wave with SDs in the REST condition before intervention (Pre), immediately after intervention (Post 0), and 15 min after intervention (Post 15).

		Non-normalized amplitude (mV)			F-wave/Mmax (F/M)			Persistence (%)		
F-wave	Pre	0.0763	±	0.0293	0.686	±	0.301	70.9	±	29.5
	Post 0	0.0769	±	0.0306	0.702	±	0.320	74.2	±	26.6
	Post 15	0.0781	±	0.0324	0.708	±	0.295	78.9	±	28.7
Mmax	Pre	12.1	±	2.5						
	Post 0	11.9	±	2.5						
	Post 15	12.0	±	2.6						

Mmax, M-wave with the maximum amplitude; SD, standard deviation.

**Fig 3:**
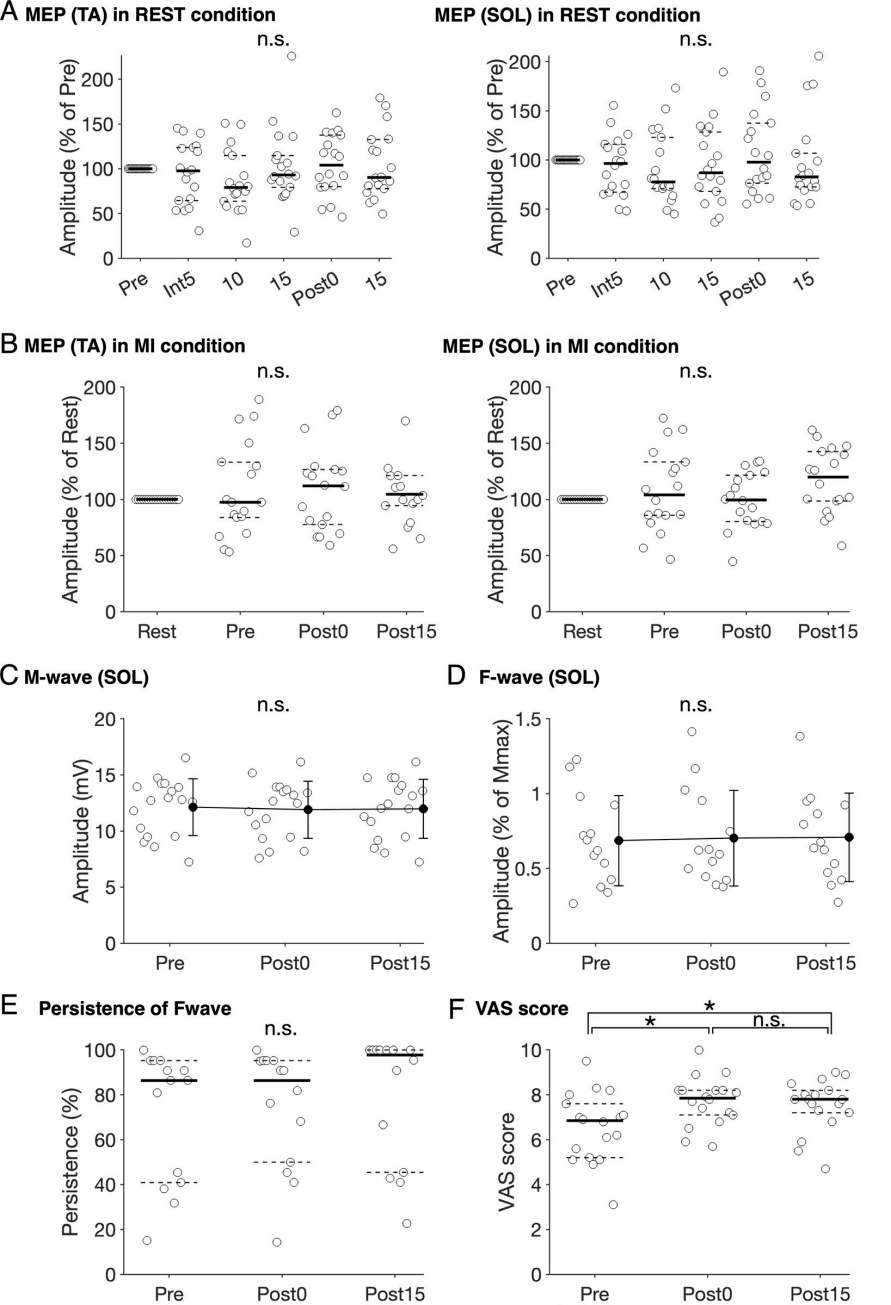
Effects of the intervention on motor evoked potential, F/M, M-wave with the maximum amplitude, and visual analogue scale scores. Changes in the average motor evoked potential (MEP) amplitudes in the tibialis anterior (TA) and soleus (SOL) muscles during the intervention (i.e., after the first block [Int5], second block [Int10], and third block [Int15]) and 0 min and 15 min after the intervention (Post0 and Post15) in the REST condition (A). Each white plot displays the average MEP amplitudes normalized as the percentage of the average MEP amplitudes before the intervention (Pre) in the REST condition (A). Changes in the average MEP amplitudes in the TA and SOL muscles before and after the intervention (pre, Post0, and Post15) in the motor imagery (MI) condition (B). Each plot displays the average MEP amplitudes normalized to the average MEP amplitudes in the REST condition (B). Solid and dashed lines represent median and interquartile ranges, respectively. Changes in the average M-wave amplitudes (C) and F/M (D) in the SOL muscle before and after the intervention in the REST condition. Black and error bars represent the mean values and SDs, respectively. Changes in persistence of F-wave before and after the intervention in the REST condition (E). Changes in visual analogue scale (VAS) scores before and after the intervention in the MI condition (F). An asterisk indicates significant differences before and 0 min after the intervention (Bonferroni-corrected p-value <0.05).

Friedman tests neither revealed significant differences in MEP in the REST condition between time points in the TA and SOL muscles (p>0.05, Table 4, Fig 3A) nor in MEP between the REST and MI conditions before intervention, 0 min, and 15 min post-intervention in the TA and SOL muscles (p>0.05, Table 4, Fig 3B). In addition, the one-way rm-ANOVA tests did not reveal significant differences in Mmax and F/M before intervention, 0 min, and 15 min post-intervention in the SOL muscle (p>0.05, Table 4, Fig 3C and 3D). Friedman tests did not reveal significant differences in F-wave persistence before intervention, 0 min, and 15 min post-intervention muscle (p>0.05, Table 4, Fig 3E).

**Table 4:** Test statistic values, p-values, effect sizes of Friedmann tests and one-way rm-ANOVA tests for the normalized MEP (% of Pre), non-normalized Mmax, F/M, and persistence of F-waves.

	Friedman tests			
	Comparison of Pre, Int5, Int10, Int15, Post0, and Post15			
MEP (TA) in REST	χ^2^ (5) = 8.254	p = 0.143	η^2^ = 0.092	n.s.
MEP (SOL) in REST	χ^2^ (5) = 6.095	p = 0.297	η^2^ = 0.068	n.s.
	Friedman tests			
** **	Comparison of REST and MI (Pre, Post0, and Post15)			
MEP (TA) in MI	χ^2^ (3) = 0.620	p = 0.982	η^2^ = 0.011	n.s.
MEP (SOL) in MI	χ^2^ (3) = 3.670	p = 0.299	η^2^ = 0.068	n.s.
	One-way rm-ANOVA tests			
** **	Comparison of Pre, Post0, and Post15			
Mmax (SOL) in REST	F (2, 34) = 0.678	p = 0.514	η^2^ = 0.038	n.s.
F/M (SOL) in REST	F (2, 26) = 0.105	p = 0.901	η^2^ = 0.008	n.s.
Persistence in REST	χ^2^ (2) = 4.978	p = 0.083	η^2^ = 0.178	n.s.

MEP, motor evoked potential; TA, tibialis anterior; MI, motor imagery; Mmax, M-wave with the maximum amplitude; ANOVA, analysis of variance; and SOL, soleus.

### 3.2. Background EMG activity

Table 5 summarizes the average normalized background EMG activity (%MVC) before TMS and PNS (peripheral nervous stimulation) (i.e., induction of MEPs and F-waves). The average MVC values with SD in the TA and SOL muscles were 0.518 ± 0.213 mV and 0.543 ± 0.156 mV, respectively. Friedman tests neither demonstrated significant differences in background EMG activity before TMS (i.e., before MEPs were induced) in the REST condition before intervention; after the first, second, and third blocks; and at 0 min and 15 min post-intervention in the TA and SOL muscles (p>0.05, Table 6) nor in the background EMG activity before TMS between the REST and MI conditions before intervention, 0 min, and 15 min post-intervention in the TA and SOL muscles (p>0.05, Table 6). Moreover, there were no significant differences in the background EMG activity before PNS (i.e., induction of F-waves and M-waves) before intervention, 0 min, and 15 min post-intervention in the SOL muscle (p>0.05, Table 6).

**Table 5:** Average normalized background EMG activity (% of MVC) with SDs in the TA and SOL muscles, 50 ms before TMS and PNS in each measurement.

50ms before TMS		Normalized background EMG (%MVC)					
		TA			SOL		
REST	Pre	0.358	±	0.191	0.398	±	0.220
	Int 5	0.406	±	0.303	0.406	±	0.222
	Int 10	0.366	±	0.219	0.376	±	0.189
	Int 15	0.372	±	0.190	0.397	±	0.214
	Post 0	0.387	±	0.260	0.446	±	0.272
	Post 15	0.344	±	0.178	0.470	±	0.290
MI	Pre	0.316	±	0.133	0.392	±	0.201
	Post 0	0.368	±	0.208	0.403	±	0.213
	Post 15	0.362	±	0.188	0.452	±	0.265
50ms before PNS		Normalized background EMG (%MVC)					
		TA			SOL		
REST	Pre	0.563	±	0.513	0.588	±	0.611
	Post 0	0.432	±	0.234	0.523	±	0.306
	Post 15	0.470	±	0.359	0.607	±	0.420

TMS, Transcranial magnetic stimulation; PNS, peripheral nervous system; TA, tibialis anterior; MI, motor imagery; EMG, electromyography; MVC, maximum voluntary contraction; SD, standard deviation; and SOL, soleus.

**Table 6:** Test statistic values, p-values, effect sizes of Friedman tests for the normalized background EMG activity (% of MVC) in the TA and SOL muscles, 50 ms before TMS and PNS.

50ms before TMS	Friedman tests			
	Comparison of Pre, Int5, Int10, Int15, Post0, and Post15			
TA in REST	χ^2^ (5) = 2.667	p = 0.751	η^2^ = 0.030	n.s.
SOL in REST	χ^2^ (5) = 6.063	p = 0.300	η^2^ = 0.067	n.s.
50ms before TMS	Friedman tests			
	Comparison of REST and MI (Pre, Post0, and Post15)			
TA in MI	χ^2^ (5) = 3.619	p = 0.605	η^2^ = 0.040	n.s.
SOL in MI	χ^2^ (5) = 10.16	p = 0.071	η^2^ = 0.113	n.s.
50ms before PNS	Friedman tests			
	Comparison of Pre, Post0, and Post15			
TA in REST	χ^2^ (2) = 0.109	p = 0.947	η^2^ = 0.017	n.s.
SOL in REST	χ^2^ (2) = 2.655	p = 0.265	η^2^ = 0.063	n.s.

TMS, Transcranial magnetic stimulation; PNS, peripheral nervous system; TA, tibialis anterior; MI, motor imagery; EMG, electromyography; and SOL, soleus.

### 3.3. VMIQ-2 and VAS scores

The average VMIQ-2 scores with SDs for external visual imagery, internal visual imagery, and kinesthetic imagery were 19.2 ± 5.3, 23.3 ± 6.1, and 25.1 ± 5.9, respectively. Spearman’s correlation analyses did not demonstrate significant correlations between the average VMIQ-2 scores and changes in MEPs and F/M (p>0.05, Table 7).

**Table 7:** Spearman correlation coefficients (r) and FDR-corrected p-values (p) between changes in MEP and VMIQ-2 scores, F/M and VMIQ-2 scores (external visual imagery, internal visual imagery, and kinesthetic imagery), and MEP and VAS scores.

		REST					MI				
		Int5	Int10	Int15	Post0	Post15	Pre	Post0		Post15	
MEP (TA) VMIQ-2 scores	External visual imagery	r = -0.261	r = -0.014	r = 0.021	r = -0.048	r = 0.105	r = -0.396	r = -0.027		r = -0.520	
		p = 0.653	p = 0.973	p = 0.973	p = 0.962	p = 0.829	p = 0.617	p = 0.973		p = 0.540	
	Internal visual imagery	r = -0.235	r = 0.259	r = 0.123	r = 0.121	r = -0.125	r = -0.612	r = -0.118		r = -0.211	
		p = 0.653	p = 0.653	p = 0.829	p = 0.829	p = 0.829	p = 0.420	p = 0.829		p = 0.706	
	Kinesthetic imagery	r = -0.092	r = -0.007	r = 0.107	r = 0.311	r = 0.108	r = -0.247	r = 0.291		r = -0.337	
		p = 0.857	p = 0.977	p = 0.829	p = 0.619	p = 0.829	p = 0.653	p = 0.631		p = 0.617	
MEP (SOL) VMIQ-2 scores	External visual imagery	r = 0.300	r = 0.443	r = 0.332	r = 0.130	r = -0.271	r = -0.409	r = -0.019		r = -0.113	
		p = 0.619	p = 0.617	p = 0.617	p = 0.829	p = 0.653	p = 0.617	p = 0.973		p = 0.829	
	Internal visual imagery	r = 0.238	r = 0.492	r = 0.235	r = 0.031	r = -0.375	r = -0.542	r = -0.189		r = 0.030	
		p = 0.623	p = 0.570	p = 0.653	p = 0.973	p = 0.617	p = 0.540	p = 0.733		p = 0.973	
	Kinesthetic imagery	r = 0.327	r = 0.247	r = 0.193	r = 0.198	r = -0.169	r = -0.301	r = 0.327		r = -0.341	
		p = 0.617	p = 0.653	p = 0.733	p = 0.733	p = 0.794	p = 0.619	p = 0.617		p = 0.617	
F/M (SOL) VMIQ-2 scores	External visual imagery				r = 0.404	r = 0.431					
					p = 0.617	p = 0.617					
	Internal visual imagery				r = 0.432	r = 0.517					
					p = 0.617	p = 0.617					
	Kinesthetic imagery				r = 0.256	r = 0.097					
					p = 0.687	p = 0.857					
MEP (TA)	VAS score						r = -0.116		r = -0.162		r = -0.264
							p = 0.829		p = 0.802		p = 0.653
							r = -0.301		r = -0.083		r = -0.427
MEP (SOL)	VAS score										
							p = 0.619		p = 0.857		p = 0.617

MI, motor imagery; MEP, motor evoked potential; TA, tibialis anterior; MI, motor imagery; VMIQ-2, Vividness of Movement Imagery Questionnaire-2; VAS, visual analogue scale; and SOL, soleus.

The average VAS score for MI of walking with SDs before intervention, 0 min, and 15 min post-intervention were 6.54 ± 1.54, 7.71 ± 1.08, and 7.51 ± 1.16, respectively. Friedman tests did not reveal significant differences in the VAS score after performing MI while walking between different time points (χ^2^ (2) = 16.20, p > 0.001, η^2^ = 0.450, Fig 3F). The VAS scores measured at 0 min and 15 min post-intervention were significantly higher than that before the intervention (before vs. 0 min post intervention, z = 3.458, Bonferroni-corrected p value = 0.002, r = 0.815; before vs. 15 min post intervention, z = 2.866, Bonferroni-corrected p value = 0.016, r = 0.657, Wilcoxon signed-rank test). There were no significant differences in the VAS scores between 0 min and 15 min post-intervention (z = 0.907, Bonferroni-corrected p-value = 1, r = 0.214, Wilcoxon signed-rank test). Spearman’s correlation analyses did not demonstrate significant correlations between MEP changes in the MI condition and VAS scores (p>0.05, Table 7).

## 4. Discussion

The present study investigated the effects of an intervention using AO+MI of walking on the corticospinal and spinal motor neuron excitability and the ability to perform MI of walking. The AO+MI intervention had no significant effect on the MEP amplitudes and F/M (p>0.05, Fig 3A and 3D). The VAS scores for MI vividness increased immediately after the intervention (p<0.05, Fig 3F), while the MEP amplitudes were stable during the MI of walking (p>0.05, Fig 3B). Therefore, the intervention using AO+MI did not modify the corticospinal and spinal motor neuron excitability; however, it temporarily improved the clarity of the MI of walking. The process had no significant effect on the M-wave amplitude (p>0.05, Fig 3C), thereby suggesting reduced or no fatigue effects. Furthermore, the average background EMG activity before TMS and PNS in each measurement was <1.5% MVC, with no significant difference between time points and conditions (p>0.05, Tables 5 and 6). Thus, fatigue effects and background EMG activity were unlikely to have affected our measurements and results.

### 4.1. No significant changes in corticospinal and spinal motoneuron excitability following intervention using AO+MI of walking

We hypothesized the facilitation of corticospinal and spinal motor neuron excitability post-intervention using AO+MI [16,18,19]. However, our present findings showed no significant facilitation of corticospinal excitability in the TA and SOL muscles following the intervention (p>0.05, Fig 3A). These results were in line with previous studies that reported no changes in corticospinal excitability after a 20-min intervention using AO+MI of hand movement and ankle dorsiflexion [33,34]. The present study is the first to investigate the effects of the intervention on the excitability of spinal motoneurons in addition to corticospinal excitability. However, our findings showed no facilitation of spinal motor neuron excitability post-intervention (p>0.05, Fig 3D).

Despite increased excitability in some participants, the changes in both types of excitability were not significantly correlated with the MI ability, evaluated with the VMIQ-2 scores (p>0.05, Table 7). Thus, the changes in excitability during and after the intervention using AO+MI were independent of this ability. A previous study reported that the MI ability was not significantly correlated with changes in corticospinal excitability at rest post-intervention using AO+MI [34]. In contrast, the MI ability was significantly correlated with changes in corticospinal excitability during MI alone, though not during AO alone or AO+MI [26,41]. Therefore, AO+MI-induced changes in corticospinal and spinal motor neuron excitability were not associated with the MI ability because the intervention also included AO components.

Although our study demonstrated that the intervention using AO+MI of walking did not significantly affect the corticospinal and spinal motor neuron excitability, rehabilitation with AO, MI, and AO+MI of walking exerts positive effects on gait improvement [5–10]. Previously, researchers provided 10- to 20-min rehabilitation sessions to participants a few times a week for more than 1 month. Therefore, it is not surprising that the single 20-min intervention using AO+MI in the present study was insufficient to induce plastic changes at the cortical and spinal levels, thereby indicating the importance of regularly scheduled interventions. Furthermore, healthy individuals may have less potential for neural plastic changes than patients with neurological disorders. Thus, it is possible that neural plastic changes did not occur because this study was conducted in healthy individuals. Another possible reason for the lack of neural plastic changes is that AO, MI, and AO+MI *per se* may exert only minor effects on neural activity while principally facilitating the effects of other interventions in rehabilitation. Previous studies showed that intervention using AO and MI modulates neural plastic changes induced by other factors [42–47]. For example, AO and MI prevent corticomotor depression induced by upper limb immobilization [42,45].

Most rehabilitation methods combine physical exercises with AO, MI, and AO+MI [6–10]. Moreover, there are hybrid approaches combining AO or MI with other strategies for rehabilitation in general [48,49]. For example, rehabilitation combining AO with dual task improved cognitive abilities in patients with Parkinson’s disease [48], while intervention of MI with structured progressive circuit class therapy increased gait ability (e.g., gait speed and stride length) in patients after stroke [49]. Therefore, AO, MI, and AO+MI might exert a marginal effect on motor improvement in rehabilitation, though they could enhance the effects of other interventions. This hypothesis is supported by the fact that other modalities, such as vibration and electrical stimulation, combined with AO, MI, or AO+MI, can induce different plastic changes in corticospinal excitability, in contrast with individual use [33,34,44,46,50]. Previous studies reported facilitated corticospinal excitability after an intervention using electrical peripheral nerve stimulation combined with AO+MI; however, there was no significant effect after using AO+MI or peripheral nerve stimulation alone in healthy individuals [33,34]. Moreover, sensory inputs induced by electrical stimulation interact with the cortical activity during AO+MI to facilitate corticospinal excitability. Therefore, despite no significant change in corticospinal and spinal motor neuron excitability following AO+MI in the present study, combining AO+MI with other modalities (e.g., electrical peripheral nerve stimulation) could induce plastic changes in excitability.

### 4.2. Increase in MI vividness without changes in corticospinal excitability during MI of walking after intervention using AO+MI

We hypothesized that the participants’ ability to perform MI of walking would increase post-intervention with AO+MI. The VAS scores measured after the MI condition significantly increased immediately and 15 min after the intervention, compared to the scores before intervention (p<0.05, Fig 3F). However, there was no significant difference in the VAS scores at 0 min and 15 min after the intervention (p>0.05, Fig 3F). Thus, the intervention temporarily improved the vividness in the MI of walking. Our results supported the aforementioned hypothesis. However, there was no significant change in the corticospinal excitability during MI of walking (p>0.05, Fig 3B). Our results suggest that the 20-min intervention using AO+MI improved the clarity of the MI of walking, though it was insufficient to facilitate the corticospinal excitability during MI.

The correlation analysis revealed that the changes in corticospinal excitability during the MI of walking were not significantly correlated with the MI ability (p>0.05, Table 7). A previous study also found no significant correlation between the vividness of MI, as determined by a questionnaire, and changes in corticospinal excitability during the MI of foot dorsiflexion and walking [17]. Other studies reported that changes in corticospinal excitability during AO+MI and MI of hand movements were significantly correlated with the MI ability assessed by the VAS [31] and VMIQ-2 scores [41]. Therefore, the association between changes in corticospinal excitability during MI and MI ability depends on the imagined tasks. For hand and simple movements, a more vivid MI imagery ability is associated with a greater change in corticospinal excitability; however, the relationship is not significant for lower-limb and complex movements, such as walking.

### 4.3. Limitations

The present study has several limitations that should be noted. First, the stimulation intensity for TMS was set to 110% RMT for evaluating the effects of the intervention on corticospinal excitability. The reason why we used this TMS setting is that a low-intensity TMS, rather than a high-intensity TMS, was recommended for studies investigating the changes in MEPs during AO [51]. However, MEPs elicited by the stimulation intensity of 110% RMT would be close to the beginning of the input-output curve and less sensitive to neural plastic changes in corticospinal excitability [52,53]. Second, lower-limb muscles may have weaker monosynaptic excitatory cortical projections than upper-limb muscles. Therefore, in addition to a low-intensity TMS, the characteristics of lower-limb corticospinal excitability might influence the MEP measurements in the present study. Third, the present study used F-waves to investigate the spinal motor neuron excitability. However, F-waves are likely to be composed of recurrent discharges of small proportion of the motor neuron pool. Thus, F-wave does not reflect the excitability of all spinal motor neurons. This might cause no significant modulation of spinal motor neuron excitability after the intervention in the present study.

## 5. Conclusion

Our results did not reveal changes in corticospinal and spinal motor neuron excitability after a 20-min intervention using AO+MI of walking. The intervention temporarily increased the vividness of MI of walking, but did not influence corticospinal excitability during MI. In other words, a short intervention using AO+MI was insufficient to induce plastic changes at the cortical and spinal levels and continually increase the MI ability in healthy individuals. Neural plastic changes may be induced by increasing the number of interventions or by combining it with other modalities. Our findings might be helpful in planning neurorehabilitation strategies for patients with neurological gait dysfunction.
